# Evaluation of Facial Soft Tissue Angles in Adolescents with Angle Class I, II, and III Malocclusion Using Profile Image Analysis

**DOI:** 10.3390/dj14060324

**Published:** 2026-05-29

**Authors:** Kristina Cernova, Andris Abeltins, Oskars Radzins, Anda Slaidina

**Affiliations:** 1 Private Practice “iDental”, LV-1001 Riga, Latvia; 2 Department of General Dentistry, Riga Stradins University, LV-1007 Riga, Latvia; andris.abeltins@rsu.lv (A.A.); anda.slaidina@rsu.lv (A.S.); 3 Institute of Stomatology, Riga Stradins University, LV-1007 Riga, Latvia

**Keywords:** facial soft tissue, facial profile, malocclusion, Angle classification, facial angles, orthodontic diagnosis, artificial intelligence, orthodontics

## Abstract

**Background/Objectives**: Soft tissue profile plays a crucial role in orthodontic diagnosis and treatment planning. However, limited data exist regarding differences in facial soft tissue angles among adolescents with different classes of malocclusion. This study aimed to evaluate variations in soft tissue facial angles among patients with Angle Class I, II, and III malocclusions aged 12–16 years using profile photographs. **Methods**: This retrospective observational study included 489 patients (330 females and 159 males; mean age 13.69 ± 1.30 years) examined between January 2008 and December 2018. 3D Slicer (Brigham and Women’s Hospital, Boston, MA, USA) was used only for landmark positioning and coordinate extraction from 2D profile photographs. Five facial angles were measured: Nasion–Nose tip–Pogonion (Na-T-Pg), Glabella–Subnasale–Pogonion (Gl-Sn-Pg), Pogonion–Nasion–Upper lip (Pg-Na-Ls), Pogonion–Nasion–Lower lip (Pg-Na-Li), and Pogonion–Subnasale–Upper lip (Pg-Sn-Ls). Statistical analysis was performed using R software, including ANOVA and *t*-tests, with significance set at *p* < 0.05. **Results**: Patients with Class III malocclusion demonstrated significantly higher mean values of the Na-T-Pg and Gl-Sn-Pg angles and lower values of the Pg-Na-Ls, Pg-Na-Li, and Pg-Sn-Ls angles compared with Class I and Class II malocclusions (*p* < 0.05), indicating mandibular protrusion. Conversely, Class II malocclusion showed lower Na-T-Pg and Gl-Sn-Pg angles and higher Pg-Na-Ls, Pg-Na-Li, and Pg-Sn-Ls values, consistent with mandibular retrusion relative to the maxilla. No clinically significant sex-related differences were observed in most parameters. **Conclusions**: Significant differences in facial soft tissue angles exist among adolescents with different malocclusion classes. These findings highlight the importance of soft tissue analysis in orthodontic diagnosis and may support the development of artificial intelligence-based tools for automated malocclusion assessment and treatment planning.

## 1. Introduction

Facial outline plays a crucial role not only in physiological activities, such as mastication, breathing, and speech, but also in communication and interpersonal relationships. Minor discrepancies in the position and size of the two sides of the face are common in the healthy population and considered normal [1].

A key factor in successful orthodontic diagnosis and treatment lies in the positioning of the soft tissues [2]. Previous studies have demonstrated that orthodontic treatment may significantly influence facial soft tissue profile characteristics and overall facial esthetics [3]. The soft tissue profile can be used to determine the treatment planning aimed at maintaining or enhancing facial esthetics. Assessing the soft tissue profile establishes the ideal size and proportions of the nose, as well as the positions of the lips and chin, which contributes to a more precise evaluation of individual facial characteristics [4].

The human nose keeps growing downward and forward at least until early adulthood, with an average annual increase in the overall external nasal length of approximately 1–1.13 mm in both males and females [5]. According to Chaconas, individuals with Class I profiles exhibit greater forward nasal tip growth compared to those with Class II profiles, while Class III individuals tend to display a more concave dorsum [6]. Nasal growth contributes substantially to age-related changes in facial profile morphology [7].

It is important to include changes in lip length and thickness associated with growth when analyzing the profile view. Notably, the lower lip grows more than the upper lip. Lip development begins earlier in girls than in boys. Growth-related changes in lip morphology also contribute to facial profile development during adolescence, with differences observed between age groups and sexes [8,9].

Variation of facial soft tissue morphology may arise from dental, skeletal, soft tissue, or functional factors, which often coexist in combination [10,11].

Craniofacial growth and development during childhood and adolescence substantially influence facial profile characteristics [12]. Previous investigations have also demonstrated that awareness and self-perception of facial soft tissue characteristics increase with age and developmental maturity [13].

Proffit and Severt reported that craniofacial characteristics vary according to the type of malocclusion present [14]. Malocclusion is recognized as the third most common oral health condition globally, with approximately 30% of the population demonstrating a significant need for orthodontic intervention [15].

Age-related mandibular growth substantially influences facial profile morphology and sagittal jaw relationships during adolescence [5,12,16].

Facial appearance and soft tissue profile characteristics play an important role in perceived facial esthetics and psychosocial well-being [17,18,19]. Severe craniofacial deformities and developmental anomalies may substantially affect facial morphology and, in some cases, require surgical correction [20,21].

Accurate orthodontic diagnosis and treatment planning are essential for achieving effective and predictable treatment outcomes [22,23]. Increasing emphasis is being placed on improving diagnostic efficiency and individualized treatment assessment in contemporary orthodontic practice.

Artificial intelligence (AI) is increasingly being investigated in orthodontics for automated image analysis, landmark identification, and treatment planning support [24,25,26,27]. Recent studies have also demonstrated the application of artificial intelligence in the analysis of facial soft tissue profile patterns and orthodontic treatment-related profile changes [28]. Deep learning approaches, including convolutional neural networks and artificial neural networks, have demonstrated promising performance in medical image analysis and pattern recognition tasks [29,30,31]. AI-based approaches may contribute to improved diagnostic standardization and workflow efficiency in future orthodontic applications [32].

Traditionally, orthodontic diagnosis was primarily based on Angle’s classification system and dental occlusion relationships. However, contemporary orthodontic treatment planning increasingly emphasizes soft tissue profile characteristics and facial esthetics in addition to skeletal and dental relationships. As a result, evaluation of facial soft tissue morphology has become an important component of modern orthodontic diagnosis and treatment planning. Recent studies have also emphasized the importance of profile harmony and facial esthetics in adolescent patients during developmental age, particularly in relation to psychosocial well-being and self-perception [33].

This study aimed to evaluate the relationship between five different facial soft tissue angles among Angle Class I, II and III malocclusions in patients aged 12 to 16 years. Accurate assessment of soft tissue profile characteristics is important for orthodontic diagnosis and treatment planning. The significance of this study is further supported by the limited data currently available regarding profile soft tissue angular relationships in adolescent patients with different malocclusion classes.

## 2. Materials and Methods

### 2.1. Study Design and Participants

This retrospective observational cohort study recruited a total of 489 healthy patients aged 12 to 16 years (mean age 13.69 ± 1.30) who were referred to the Department of Orthodontics, Institute of Stomatology, between January 2008 and December 2018.

Patients with Class I, II, and III malocclusion who were in mid-puberty received orthodontic consultation to create an appropriate treatment plan. All patients were examined by experienced orthodontists, while dental photos were provided by two professional photographers who took all intraoral and extraoral photos. The photos were taken according to a single standardized protocol. For this study, it was decided to use only extraoral profile photos.

### 2.2. Ethical Consideration and Study Registration

This study was performed in accordance with the guidelines of the Ethics Committee of Rīga Stradiņš University (RSU). The study was conducted according to the principles of the Declaration of Helsinki (No. 2-PĒK-4/748/2025).

No identifiable facial images are presented. Schematic diagrams were created solely for methodological illustration.

### 2.3. Inclusion and Exclusion Criteria

This study included all patients aged 12 to 16 years (including 16-year-old children) with no history of trauma.

The exclusion criteria were as follows: patients who were under 12 years old and over 16 years old; patients with craniofacial anomalies or syndromes; patients with cleft lip or cleft palate; patients with beards and/or mustaches; patients with long bangs that overlay the lower third of the forehead; patients who had previously undergone orthodontic treatment; and patients who had undergone orthognathic or maxillofacial surgery.

Soft tissue profile characteristics were evaluated in relation to the patient’s skeletal malocclusion class.

### 2.4. Image Acquisition

**Figure 1:**
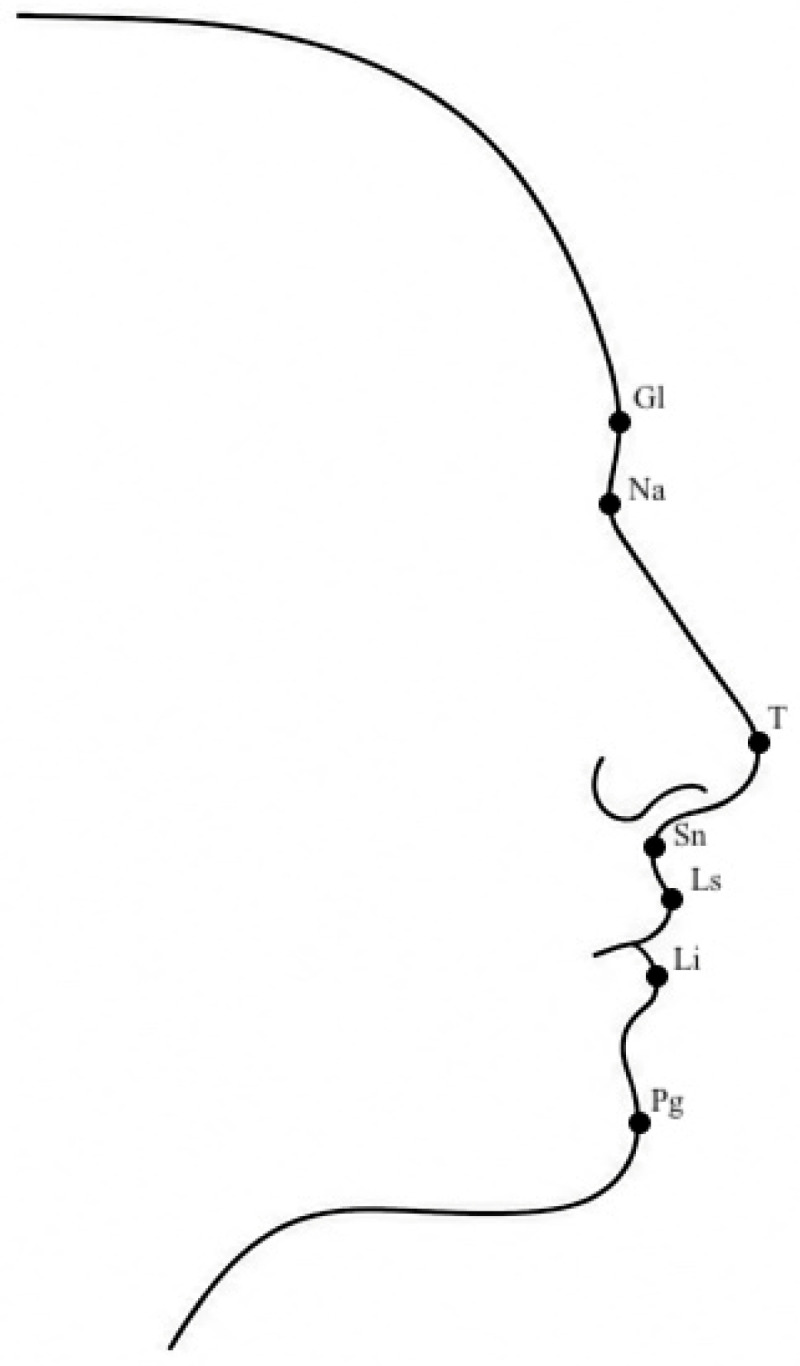
Schematic representation of anthropometric landmarks in the profile view. The same landmarks were consistently used for all subjects. Abbreviations: Gl—glabella; Na—nasion; T—nose tip; Sn—subnasale; Ls—labium superior; Li—labium inferior; and Pg—soft tissue pogonion.

**Figure 2:**
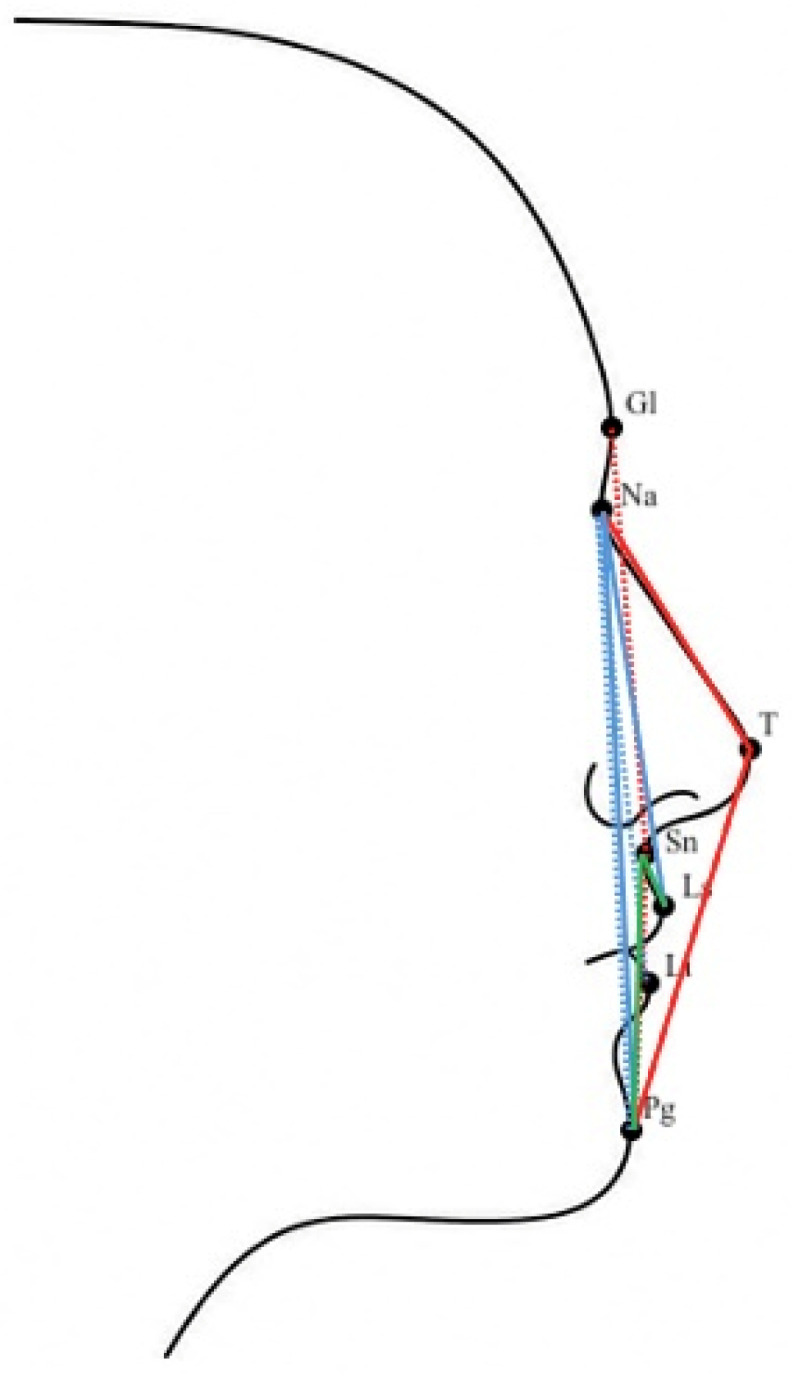
Schematic illustration of the five facial soft tissue angular measurements used in the study: Na-T-Pg, Gl-Sn-Pg, Pg-Na-Ls, Pg-Na-Li, and Pg-Sn-Ls. The colored lines represent the schematic visualization of the angular measurements and are used only for illustration purposes. Solid and dashed red lines indicate Na-T-Pg and Gl-Sn-Pg measurements; solid and dashed blue lines indicate Pg-Na-Ls and Pg-Na-Li measurements; green lines indicate Pg-Sn-Ls measurement.

All profile photographs were acquired by professional photographers according to a standardized photographic protocol. Patients were positioned with a neutral facial expression and without occlusal contact between the teeth. Images with poor focus, motion artifacts, inadequate image quality or incorrect head positioning were excluded from the study. Seven anthropometric landmarks were identified on each profile photograph and five facial soft tissue angles were measured: Nasion–Nose tip–Pogonion (Na-T-Pg), Glabella–Subnasale–Pogonion (Gl-Sn-Pg), Pogonion–Nasion–Upper lip (Pg-Na-Ls), Pogonion–Nasion–Lower lip (Pg-Na-Li), and Pogonion–Subnasale–Upper lip (Pg-Sn-Ls). The corresponding landmarks and angular measurements are illustrated schematically in Figure 1 and Figure 2. After acquisition, the images were analyzed using 3D Slicer (Brigham and Women’s Hospital, Boston, MA, USA; https://www.slicer.org) software for digital landmark positioning and coordinate extraction from two-dimensional profile images. The 3D Slicer platform was used as a digital image analysis tool for two-dimensional landmark positioning and coordinate extraction from profile photographs.

No identifiable facial images are presented in this manuscript. For methodological illustration, all original photographs shown in the figures were replaced with schematic diagrams demonstrating the anthropometric landmarks and angular measurements used in the study. These schematic illustrations do not correspond to real individuals. Following image acquisition, profile photographs were analyzed using the 3D Slicer platform for landmark positioning and coordinate extraction.

### 2.5. Quantification of Image with Landmarks

Seven anthropometric landmarks were manually identified on each profile photograph by a single operator: Gl (glabella), Na (nasion), T (nose tip), Sn (subnasale), Ls (labium superior), Li (labium inferior), and Pg (soft tissue pogonion). Following landmark placement, the x- and y-coordinate positions were extracted using the 3D Slicer platform.

The anatomical locations of these landmarks are illustrated schematically in Figure 1 and Figure 2.

### 2.6. Reproducibility and Error of Method Analysis

The first step of this study was to assess the intra-operator repeatability of facial anthropometric landmark identification on two-dimensional profile photographs. A single operator performed all landmark identifications and measurements. Twenty randomly selected profile photographs were re-evaluated after a two-week interval to assess the consistency of landmark placement. A subset of 20 images was considered sufficient for intra-operator reproducibility assessment and was consistent with previously published methodological studies involving repeated landmark identification. The intraclass correlation coefficient (ICC) was calculated to evaluate measurement reliability. The mean ICC value of 0.999 indicated excellent reproducibility of landmark identification and angular measurements. Landmark coordinates were extracted from the software and recorded in a Microsoft Excel spreadsheet before statistical analysis. The R scripts used for angle calculations are available upon reasonable request.

### 2.7. Statistical Analyses

All data were statistically analyzed using R version 3.6.1 (R Foundation for Statistical Computing, Vienna, Austria; https://www.r-project.org/). The ANOVA test was used to analyze differences among all five angles of Class I, II, and III malocclusions. A *t*-test was used to compare each of the five facial angles between two different malocclusion groups. A *p*-value < 0.05 was considered statistically significant. Microsoft Excel was used to calculate the mean age and each angle in degrees for females and males.

## 3. Results

Four hundred eighty-nine patients with dentoalveolar Class I, II, and III were included in the study (Table 1). Overall, this study included 330 females (mean age 13.66 ± 1.27) and 159 males (mean age 13.74 ± 1.37) classified as Class I, II, or III (Table 1). In total, the study included 130 patients (mean age 13.72 ± 1.28 SD) with Class I malocclusion, 314 patients (mean age 13.65 ± 1.31 SD) with Class II malocclusion, and 45 patients (mean age 13.84 ± 1.35 SD) with Class III malocclusion. The largest study group consisted of females with Angle Class II malocclusion (212 patients, mean age 13.60 ± 1.30 SD), while the smallest group consisted of males with Angle Class III malocclusion (22 patients, mean age 13.64 ± 1.64 SD).

### 3.1. Angle Characteristics

All five facial angles demonstrated statistically significant differences among malocclusion classes (*p* < 0.05) based on ANOVA analysis (Table 2). Pairwise comparisons using *t*-tests also revealed statistically significant differences between Class I and II, Class I and III, and Class II and III malocclusions. Patients with Class III malocclusion showed the highest mean values of the Na-T-Pg and Gl-Sn-Pg angles and lowest values of the Pg-Na-Ls, Pg-Na-Li and Pg-Sn-Ls angles, indicating a tendency toward mandibular protrusion. Previous studies evaluating skeletal Class III malocclusion have similarly reported that orthodontic treatment-related changes in soft tissue profile characteristics may contribute to improved facial profile harmony and esthetic balance [34]. These findings suggest that soft tissue angular measurements may reflect characteristic sagittal profile differences associated with mandibular positioning among different malocclusion classes. In contrast, Class II malocclusion demonstrated lower Na-T-Pg and Gl-Sn-Pg values and higher Pg-Na-Ls, Pg-Na-Li and Pg-Sn-Ls values, consistent with mandibular retrusion relative to the maxilla. The Pg-Na-Li angle showed similar values in Class I and Class II malocclusions but lower values in Class III cases, suggesting similarities in facial profile characteristics between Class I and Class II patients.

### 3.2. Comparing Males and Females

No statistically significant differences were found between the sexes in any of the five facial angles across all malocclusion classes (Table 3). Female participants, overall, had a higher mean value than males in all facial angles except for the Pg-Na-Li, where the result was 4.29 ± 1.94 SD for females and 4.41 ± 1.97 SD for males, although this difference was not statistically significant. The Class II results were similar to the overall totals in both groups, due to the higher prevalence of Class II patients. A *t*-test comparing males and females for Class II (0.0003) and the total sample (0.01) yielded statistically significant results.

### 3.3. Landmark Accuracy

To assess landmark positioning reliability, seven anthropometric landmarks were re-evaluated on profile photographs of 20 randomly selected patients after a two-week interval. The intraclass correlation coefficient (ICC) demonstrated excellent intra-operator reproducibility, with a mean ICC value of 0.999 across all landmarks and angular measurements. The mean difference between repeated landmark placements was less than 0.5 mm.

## 4. Discussion

The purpose of this study was to collect data and evaluate the differences in the soft tissue angles among patients with Class I, Class II, and Class III malocclusions, using profile images of individuals aged 12 to 16 years.

A notable strength of the present study is the relatively large sample size (n = 489), which increases the statistical power of the analysis and improves the reliability of the observed differences among malocclusion classes.

At the beginning of 2024, Latvia had a population of 1,872,000, of which children from 12 to 16 (inclusive) years old were 5.4% of the population. Out of all children, males were 2.8% of the population and females were 2.6% of the population at the age from 12 to 16 inclusive [35].

As mentioned before, craniofacial soft tissue characteristics may be influenced by dental, skeletal, soft tissue, and functional issues [10,11,14]. Variations in facial profile morphology are commonly observed among individuals with different malocclusion classes and may influence facial esthetics and psychosocial perception, particularly during adolescence and young adulthood. The findings of the present study demonstrate significant differences in soft tissue angular measurements among Class I, II, and III malocclusions, emphasizing the importance of precise profile assessment in orthodontic diagnosis and treatment planning. The observed profile variations among malocclusion groups further highlight the complexity of craniofacial growth and the importance of individualized orthodontic evaluation.

Longitudinal studies on postpubertal growth are limited; nevertheless, findings from a study of 45 Danish males revealed a mandibular growth of about 3 mm between the ages of 16 and 17 [36].

Growth-related changes in craniofacial anatomy play a critical role in shaping the soft tissue profile during adolescence—the key age group evaluated in this study. Foley and Mamandras observed that overall mandibular growth was approximately twice as great as overall maxillary growth. The mandibular growth rate between the ages of 14 and 16 was found to be twice as rapid as during later years. During this time, the posterior vertical dimension of the face increased slightly more than the anterior dimension. Additionally, the mandibular plane angle decreased by 1.1 degrees between the ages of 14 and 20 years, causing the possibility of a closing rotation of the mandible. With advancing age, the mandibular incisors also appeared to tip labially [37]. These developmental changes are particularly relevant to the present study, as they directly influence the soft tissue profile, especially in patients with Class II and Class III malocclusions, where mandibular positioning is a key factor.

Similarly, the mandible of young children is relatively small and retrusive compared to the maxilla, and the chin is incompletely formed in the infant. As growth continues, the chin tends to shift forward relative to the upper facial skeleton, with the mandibula transitioning from a more retruded to a less retruded position [12]. This developmental pattern is particularly relevant to the current study when evaluating soft tissue angles such as the Na-T-Pg and Gl-Sn-Pg, which reflect the sagittal positioning of the mandible in relation to the upper face.

In addition to sagittal and vertical changes, transverse arch development also influences facial appearance. Research indicates that in the maxillary arch intercanine width increases by approximately 6 mm and the intermolar width by about 2 mm by the age of 13. In the mandibular arch, the intercanine width expands by 3.7 mm up to age 13, while the intermolar width increases by 1.5 mm between ages 3 and 5 and by an additional 1 mm from ages 8 to 13 [38]. These changes affect the underlying dental and skeletal support for soft tissue, contributing to the overall profile contour assessed in this study. Understanding such growth dynamics is essential for interpreting age-related variations in facial angles and for improving the accuracy of orthodontic diagnosis and treatment planning during this critical period of craniofacial development.

Previous studies have emphasized the importance of soft tissue evaluation in orthodontic diagnosis and facial esthetic assessment [14,39]. However, many investigations have primarily focused on hard tissue structures, while comparatively fewer studies have analyzed soft tissue profile characteristics. Particular attention has been directed toward the nasal and perioral regions, as visual perception studies suggest that observers initially focus on the central facial region when evaluating facial appearance [40].

The facial surface is the most visible area that clinicians and lay people appreciate and often forms the basis of aesthetic evaluations. Understanding the external soft tissue integument and its relationship with the underlying hard tissue skeleton is important, particularly with the increasing emphasis on soft tissue considerations in various medical fields [41]. There are other facial angles which provide different characteristics for each malocclusion. The nasolabial angle becomes more acute due to the decreased lip prominence and lowered nasal tip. The descent and rotation of the nasal tip are accompanied by corresponding lip movement, a process referred to as a clockwise rotation of the nasolabial complex. This rotation, along with a slight decrease in the mentolabial angle, is observed between the ages of 7 and 18 years in both sexes [42]. Some individuals’ facial structure may appear less protrusive with age due to several factors, including the continued uprighting of the maxillary incisors during adulthood, nasal growth, lip repositioning, and vertical facial height increase. These changes collectively contribute to a less protrusive facial appearance over time [12].

The present dataset of facial soft tissue angular measurements may provide a useful foundation for future artificial intelligence (AI)-based investigations in orthodontics. AI-driven approaches could potentially assist in automated landmark identification, facial profile analysis, and predictive modeling in future studies involving larger and more diverse datasets. However, the current study did not directly apply machine learning or AI-based analytical methods.

This study shows how the surface-based method was used for soft tissue analysis. The method is comparatively fast, simple, and landmark-independent and is a valuable diagnostic and treatment planning tool not only for orthognathic but also for cosmetic surgery patients with soft-tissue asymmetry. Numerous studies have demonstrated that two-dimensional (2D) peri-apical (PA) cephalometric radiographs offer limited reliability and diagnostic value in orthodontic treatment planning, due to the superimposition of deeper craniofacial bony structures and the inadequate visualization of soft tissues [41,43].

Historically, two-dimensional (2D) photographic analysis has complemented clinical orthodontic examination by enabling the evaluation of facial soft tissue profile characteristics and craniofacial relationships [44]. However, two-dimensional measurements may be influenced by factors such as head positioning, image quality, and landmark identification accuracy; therefore, the use of stable anatomical reference points and standardized photographic protocols is important for improving measurement reliability and reproducibility [45].

The comprehensive statistical analyses conducted in this study have allowed valuable insights into facial angles between different malocclusions. Importantly, the absence of statistically significant differences between males and females in facial angles suggests that treatment approaches may not need to be sex-specific; however, the allure of facial aesthetics shared by both genders is a crucial consideration in treatment planning. The higher proportion of female participants with Class II malocclusion observed in the present study may reflect the distribution of patients referred for orthodontic evaluation during the study period.

In addition to the retrospective design and unequal subgroup distribution, the present study has several limitations. The unequal subgroup distribution reflects the natural prevalence of malocclusion classes within the examined population and should be considered when interpreting the statistical comparisons among groups. In addition, multiple pairwise comparisons were performed without formal adjustment for multiple testing, which should be considered when interpreting marginally significant findings. Anthropometric landmark placement was performed manually by a single operator, which may have introduced minor operator-dependent variability despite the excellent intra-operator reproducibility observed. Furthermore, the study was limited to two-dimensional profile image analysis and did not include three-dimensional soft tissue evaluation. The retrospective observational design may also carry a risk of selection bias, particularly due to the exclusion of patients with craniofacial anomalies or previous orthodontic treatment. Future longitudinal studies involving larger and more diverse populations may provide additional insight into age-related changes in facial soft tissue profile characteristics. In addition, the present dataset may provide a foundation for future artificial intelligence-based investigations involving automated facial profile analysis and predictive orthodontic modeling.

## 5. Conclusions

This study demonstrated that significant differences in soft tissue facial angles exist among patients with Class I, Class II, and Class III malocclusions in the 12 to 16-year-old age group. Specifically, Class III malocclusion was associated with increased Na-T-Pg and Gl-Sn-Pg angles, indicating a more pronounced mandibular protrusion, while Class II malocclusion showed the opposite trend, reflecting mandibular retrusion. The high intra-operator reliability confirms the consistency of the landmark placement, validating the use of two-dimensional profile photographs for soft tissue analysis. Importantly, the angle data collected in this study may provide a useful dataset for future artificial intelligence-based investigations involving automated facial profile assessment and orthodontic diagnostic support.

## Figures and Tables

**Table 1:** Selected data and distribution of patients between males and females (*N* = 489).

Age (Years)		Range				12–16
	Male		Female		All	
Class	Count	Age (Mean ± SD)	Count	Age (Mean ± SD)	Count	Age (Mean ± SD)
I	35	13.63 ± 1.33	95	13.76 ± 1.27	130	(13.72 ± 1.28)
II	102	13.75 ± 1.33	212	13.60 ± 1.30	314	(13.65 ± 1.31)
III	22	13.64 ± 1.64	23	13.83 ± 1.03	45	(13.84 ± 1.35)
Total	159	13.74 ± 1.37	330	13.66 ± 1.27	489	(13.69 ± 1.30)

Abbreviations: *N*: number of patients. SD: standard deviation. I: Angle Class I malocclusion of first molar relationship. II: Angle Class II malocclusion of first molar relationship. III: Angle Class III malocclusion of first molar relationship.

**Table 2:** Descriptive statistics and comparisons of mean degrees of all five facial angles between different malocclusions: Class I, Class II, and Class III.

					*p*-Value
Class	Na-T-Pg	Gl-Sn-Pg	Pg-Na-Ls	Pg-Na-Li	Pg-Sn-Ls
	(Mean ± SD)	(Mean ± SD)	(Mean ± SD)	(Mean ± SD)	(Mean ± SD)
I	128.24 ± 4.08	169.42 ± 4.75	6.33 ± 2.14	4.05 ± 1.60	10.66 ± 5.95
II	125.89 ± 4.59	165.75 ± 5.22	7.72 ± 2.43	4.61 ± 2.02	13.16 ± 6.43
III	131.98 ± 5.06	173.34 ± 4.68	3.88 ± 2.21	3.24 ± 1.86	7.08 ± 5.28
Total	127.07 ± 4.87	167.42 ± 5.61	6.99 ± 2.60	4.33 ± 1.94	11.93 ± 6.48
^a^ ANOVA	*p* < 0.0001	*p* < 0.0001	*p* < 0.0001	*p* < 0.0001	*p* < 0.0001
^b^ *t*-test					
I vs. II	2.165 × 10^−7^ *	6.456 × 10^−12^ *	6.861 × 10^−9^ *	0.002146 *	0.0001083 *
I vs. III	0.00003097 *	0.000007028 *	1.007 × 10^−8^ *	0.01121 *	0.0002857 *
II vs. III	3.522 × 10^−10^ *	1.1746× 10^−14^ *	1.194 × 10^−15^ *	0.00002525 *	1.794 × 10^−9^ *

Abbreviations: ^a^ ANOVA test for comparisons among all malocclusion classes. ^b^ *t*-test for pairwise comparisons between malocclusion groups. * *p* < 0.05 considered statistically significant. I: Angle Class I malocclusion of first molar relationship. II: Angle Class II malocclusion of first molar relationship. III: Angle Class III malocclusion of first molar relationship. Nasion–Nose tip–Pogonion (Na-T-Pg), Glabella–Subnasale–Pogonion (Gl-Sn-Pg), Pogonion–Nasion–Upper lip (Pg-Na-Ls), Pogonion–Nasion–Lower lip (Pg-Na-Li), and Pogonion–Subnasale–Upper lip (Pg-Sn-Ls).

**Table 3:** Descriptive statistics and comparisons of mean degrees of all five facial angles between different malocclusions: Class I, Class II, and Class III in males and females.

				*p*-Value
Face Angles	Class I	Class II	Class III	Total
Na-T-Pg				
Males	129.15 ± 4.42	125.08 ± 4.87	132.63 ± 5.69	127.02 ± 5.61
Females	127.90 ± 3.91	126.27 ± 4.42	131.36 ± 4.42	127.10 ± 4.48
** *t*-test	0.147	0.0383 *	0.411	0.884
Gl-Sn-Pg				
Males	169.05 ± 4.16	164.20 ± 5.37	172.79 ± 5.05	166.45 ± 5.99
Females	169.56 ± 4.96	166.50 ± 4.99	173.86 ± 4.34	167.89 ± 5.37
** *t*-test	0.5625	0.0003577 *	0.4482	0.01072 *
Pg-Na-Ls				
Males	5.80 ± 1.89	8.07 ± 2.53	3.90 ± 2.41	6.99 ± 2.83
Females	6.52 ± 2.20	7.55 ± 2.36	3.87 ± 2.05	7.00 ± 2.49
** *t*-test	0.07148	0.08303	0.9561	0.9926
Pg-Na-Li				
Males	3.81 ± 1.35	4.87 ± 2.01	3.25 ± 1.90	4.41 ± 1.97
Females	4.13 ± 1.68	4.48 ± 2.01	3.23 ± 1.64	4.29 ± 1.94
** *t*-test	0.2559	0.103	0.9734	0.5156
Pg-Sn-Ls				
Males	8.93 ± 4.90	13.04 ± 6.51	6.93 ± 5.68	11.29 ± 6.51
Females	11.29 ± 6.19	13.21 ± 6.41	7.23 ± 4.99	12.24 ± 6.45
** *t*-test	0.02639 *	0.8275	0.8532	0.1298

Abbreviations: * Statistically significant: *p* < 0.05. ** *t*-test *p*-value comparing males vs. females (same class). SD: standard deviation. I: Angle Class I malocclusion of first molar relationship. II: Angle Class II malocclusion of first molar relationship. III: Angle Class III malocclusion of first molar relationship. Nasion–Nose tip–Pogonion (Na-T-Pg), Glabella–Subnasale–Pogonion (Gl-Sn-Pg), Pogonion–Nasion–Upper lip (Pg-Na-Ls), Pogonion–Nasion–Lower lip (Pg-Na-Li), and Pogonion–Subnasale–Upper lip (Pg-Sn-Ls).

## Data Availability

Data are available upon reasonable request from the corresponding author (K. Cernova) due to privacy restrictions.

## References

[1] Farkas L.G. (1994). Anthropometry of the Head and Face.

[2] Baysal A., Sahan A.O., Ozturk M.A., Uysal T. (2016). Reproducibility and Reliability of Three-Dimensional Soft Tissue Landmark Identification. Angle Orthod..

[3] Moon S., Mohamed A.M.A., He Y., Dong W., Chen Y., Yang Y. (2021). Extraction vs. Nonextraction on Soft-Tissue Profile Change in Patients with Malocclusion: A Systematic Review and Meta-Analysis. BioMed Res. Int..

[4] Bergman R.T., Waschak J., Borzabadi-Farahani A., Murphy N.C. (2014). Longitudinal Study of Cephalometric Soft Tissue Profile Traits. Angle Orthod..

[5] Sharma P., Arora A., Valiathan A. (2014). Age Changes of Jaws and Soft Tissue Profile. Sci. World J..

[6] Chaconas S.J. (1969). A Statistical Evaluation of Nasal Growth. Am. J. Orthod..

[7] Wisth P.J. (2007). Changes of the Soft Tissue Profile During Growth. Eur. J. Orthod..

[8] Subtelny J.D. (1959). A Longitudinal Study of Soft Tissue Facial Structures and Their Profile Characteristics, Defined in Relation to Underlying Skeletal Structures. Am. J. Orthod..

[9] Vig P.S., Cohen A.M. (1979). Vertical Growth of the Lips: A Serial Cephalometric Study. Am. J. Orthod..

[10] Bergeron L., Tu C.C., Chen Y.R. (2008). Single-Split Technique for Correction of Severe Facial Asymmetry. Plast. Reconstr. Surg..

[11] Cheong Y.W., Lo L.J. (2011). Facial Assymetry: Etiology, Evaluation and Management. Chang Gung Med. J..

[12] Enlow D.H., Hans M.G. (2008). Handbook of Facial Growth.

[13] Varatharaju V., Caflisch M., Soroken C., Kiliaridis S., Antonarakis G.S. (2021). Does Age Influence Self-Perception of the Soft-Tissue Profile in Children?. Am. J. Orthod. Dentofac. Orthop..

[14] Proffit W., Severt T. (1997). The Prevalence of Facial Asymmetry in the Dentofacial Deformities Population at the University of North Carolina. Int. J. Adult Orthodon. Orthognath. Surg..

[15] Cenzato N., Nobili A., Maspero C. (2021). Prevalence of Dental Malocclusions in Different Geographical Areas. Dent. J..

[16] Björk A., Palling M. (1955). Adolescent Age Changes in Sagittal Jaw Relation. Acta Odontol. Scand..

[17] Grammer K., Thornhill R. (1994). Human Facial Attractiveness and Sexual Selection. J. Comp. Psychol..

[18] Jones D., Hill K. (1993). Criteria of Facial Attractiveness in Five Populations. Hum. Nat..

[19] Shaw W.C., Rees G., Dawe M., Charles C.R. (1985). The Influence of Dentofacial Appearance on the Social Attractiveness of Young Adults. Am. J. Orthod..

[20] Eubanks R.J. (1957). Surgical Correction of Masseter Muscle Hypertrophy Associated with Unilateral Prognathism: Report of case. J. Oral Surg..

[21] Kronmiller J.E. (1998). Development of Asymmetries. Semin. Orthod..

[22] Aljehani D., Baeshen H.A. (2018). Effectiveness of the American Board of Orthodontics Discrepency Index in Predicting Treatment Time. J. Contemp. Dent. Pract..

[23] Shimizu Y., Tanikawa C., Kajiwara T., Nagahara H., Yamashiro T. (2022). The Validation of Orthodontic Artificial Intelligence Systems that Perform Orthodontic Diagnoses and Treatment Planning. Eur. J. Orthod..

[24] Albalawi F., Alamoud K.A. (2022). Trends and Application of Artificial Intelligence Technology in Orthodontic Diagnosis and Treatment Planning—A Review. Appl. Sci..

[25] Kulikowski C.A. (2015). An Opening Chapter of the First Generation of Artificial Intelligence in Medicine. Med. Inform..

[26] Li Z., Liu F., Yang W., Peng S., Zhou J. (2022). A Survey of Convolutional Neural Networks: Analysis, Applications, and Prospects. IEEE Trans. Neural Netw. Learn. Syst..

[27] Liu J., Zhang C., Shan Z. (2023). Application of Artificial Intelligence in Orthodontics: Current state and Future Perspectives. Healthcare.

[28] Tanikawa C., Tan T.J., Takada K. (2024). Facial Soft-Tissue Shape Changes after Fixed Edgewise Treatment with Premolar Extraction in Individual Artificial-Intelligence-Classified Facial Profile Patterns. BMC Oral Health.

[29] Sharif M.S., Abbod M., Amira A., Zaidi H. (2010). Artificial Neural Network-Based System for PET Volume Segmentation. Int. J. Biomed. Imaging.

[30] Tomè D., Monti F., Baroffio L., Bondi L., Tagliasacchi M., Tubaro S. (2016). Deep Convolutional Neural Networks for Pedestrian Detection. Signal Process. Image Commun..

[31] Wang X.L., Liu J., Li Z.Q., Luan Z.L. (2021). Application of Physical Examination Data on Health Analysis and Intelligent Diagnosis. BioMed. Res. Int..

[32] Monilla-Gonzalez A., Rovira-Calatayud L., Gustavo d’Oliveira N., Ustrell-Torrent J.M. (2021). Artificial Intelligence in Orthodontics: Where are we now? A scoping Review. Orthod. Craniofac. Res..

[33] Parisi G.M., Partipilo E., Gisondi C., Condrò P., Anastasio M.D., Brescia A.V., Docimo R. (2024). Aesthetic Perception of Patient in Developmental Age in Interceptive Orthodontic Treatment. Eur. J. Paediatr. Dent..

[34] Liu L., Liu Y., Guo K., Ma H., Yang F. (2025). Soft and Hard Tissue Changes after Compensatory Treatment in Skeletal Class III Malocclusion. PLoS ONE.

[35] (2024). Children in Latvia Collection of Statistics. https://admin.stat.gov.lv/system/files/publication/2024-08/Nr_05_Berni_Latvija_2024_%2824_00%29_LV_EN.pdf.

[36] Björk A. (1963). Variations in the Growth Pattern of the Human Mandible. J. Dent. Res..

[37] Foley T.F., Mamandras A.H. (1992). Facial Growth in Females 14 to 20 Years of Age. Am. J. Orthod. Dentofac. Orthop..

[38] Bishara S.E., Jakobsen J.R., Treder J., Nowak A. (1997). Arch Width Changes from 6 Weeks to 45 Years. Am. J. Orthod. Dentofac. Orthop..

[39] Chew M.T. (2006). Spectrum and Management of Dentofacial Deformities in a Multiethnic Asian Population. Angle Orthod..

[40] Meyer-Marcotty P., Alpers G.W., Gerdes A.B.M., Stellzig-Eisenhauer A. (2010). Impact of Facial Asymmetry in Visual Perception: A 3-dimensional data analysis. Am. J. Orthod. Dentofac. Orthop..

[41] Patel A., Islam S.M.S., Murray K., Goonewardene M.S. (2015). Facial Asymmetry Assessment in Adults Using 3D Imaging. Prog. Orthod..

[42] Nanda R.S., Meng H., Kapila S., Goorhuis J. (1990). Growth Changes in the Soft Tissue Facial Profile. Angle Orthod..

[43] Shah S.M., Joshi M.R. (1978). An Assessment of Asymmetry in the Normal Craniofacial Complex. Angle Orthod..

[44] de Moraes M.E., Hollender L.G., Chen C.S., Moraes L.C., Balducci I. (2011). Evaluating Craniofacial Asymmetry with Digital Cephalometric Images and Cone-beam Computed Tomography. Am. J. Orthod. Dentofac. Orthop..

[45] Miller L., Morris D.O., Berry E. (2007). Visualizing Three-Dimensional Facial Soft-Tissue Changes Following Orthognathic Surgery. Eur. J. Orthod..

